# Preliminary Report on Intestinal Flora Disorder, Faecal Short-Chain Fatty Acid Level Decline and Intestinal Mucosal Tissue Weakening Caused by Litchi Extract to Induce Systemic Inflammation in HFA Mice

**DOI:** 10.3390/nu14040776

**Published:** 2022-02-12

**Authors:** Dongfang Sun, Chen Wang, Lijun Sun, Lianhua Hu, Zhijia Fang, Qi Deng, Jian Zhao, Ravi Gooneratne

**Affiliations:** 1 Guangdong Provincial Key Laboratory of Aquatic Product Processing and Safety, College of Food Science and Technology, Guangdong Ocean University, Zhanjiang 524088, China; dongfang_gdou@163.com (D.S.); 18642326533@163.com (C.W.); lianhuashipin@126.com (L.H.); fzj4437549@163.com (Z.F.); dengqi1024@163.com (Q.D.); 2 School of Chemical Engineering, The University of New South Wales, Sydney, NSW 2052, Australia; jian.zhao@unsw.edu.au; 3 Department of Wine, Food and Molecular Biosciences, Faculty of Agriculture and Life Sciences, Lincoln University, Canterbury 7647, New Zealand; ravi.gooneratne@lincoln.ac.nz

**Keywords:** gut microbiota, systemic inflammation, litchi

## Abstract

Certain foods are known as “heating” foods in Chinese medicine. Over-consumption of these foods can lead to symptoms known as “heating up”. These symptoms have been shown to be symptoms of systemic low-grade inflammation. However, the mechanism by which these foods cause inflammation is not clear. In this preliminary study, we investigated dysbacteriosis of the gut microbiota as a possible cause of inflammation by litchi, a typical “heating” food. A human flora-associated (HFA) mouse model (donor: n = 1) was constructed. After gavaging the mice with litchi extract suspension at low, medium and high doses (400, 800, 1600 mg/kg·d^−1^, respectively) (n = 3) for 7 days, the serum levels of inflammatory cytokines, gut microbiota, the concentration of SCFAs and the integrity of the intestinal mucosal barrier were measured. The results revealed significant increases in the abundance of *Prevotella* and *Bacteroides*. A significant increase in the abundance of *Bilophila* and a decrease in *Megasomonas* was observed in the high-dose group. High-dose litchi intervention led to a decrease of most SCFA levels in the intestine. It also caused a more than two-fold increase in the serum TNF-α level and LPS level but a decrease in the IL-1β and IL-6 levels. Medium- and high-dose litchi intervention caused widening of the intestinal epithelial cell junction complex and general weakening of the intestinal mucosal barrier as well as reduced energy conversion efficiency of the gut microbiota. These data suggest that litchi, when consumed excessively, can lead to a low degree of systematic inflammation and this is linked to its ability to cause dysbacteriosis of the gut microbiota, decrease SCFAs and weaken the intestinal mucosal tissues.

## 1. Introduction

Certain foods, such as litchi, longan, mango, durian, orange, chili, pepper, etc., are known as “heating” foods in traditional Chinese medicine. Excessive consumption of “heating” foods may cause a number of disorders, such as red and swollen eyes, acne, sores and ulcers in the mouth and tongue, swollen gums, sore throat, yellow urine, constipation and other symptoms; these symptoms are known as “shanghuo” (heating-up) in Chinese medicine [1,2,3]. Studies have shown that “heating” foods such as litchi and citrus cause “shanghuo”, mainly through systemic low-grade inflammatory reactions caused by several macromolecular substances in these foods; however, the exact mechanism is not clear [4,5,6]. Systemic low-grade inflammation is a non-specific and persistent pathological state of inflammation in the body. It is mainly manifested as significantly elevated levels of inflammatory markers in the blood, such as TNF-α, and this can last for a long period of time [7]. In recent years, many studies have shown that systemic low-grade inflammation has a direct causal relationship with the occurrence and development of many important chronic diseases in humans, including cancer, diabetes, liver diseases, inflammatory bowel disease, irritable bowel syndrome, allergies, asthma, autism, depression, Alzheimer’s disease and aging [7,8,9]. It has become one of the focal issues in the field of medicine, health and nutrition.

There are over 1000 species of microorganisms in the gut flora, containing more than 100 times as many genes as the human body [10]. Thus, the gut microbiota is dubbed “the body’s second gene pool” and is believed to be vital to human health [7]. In recent years, due to breakthroughs in molecular biology techniques for studying the gut microbiota, the relationship between the gut microbiota and systemic low-grade inflammatory chronic diseases has received increased research attention. Previous etiological studies have demonstrated that gut microbiota disorders are among the most important driving forces underlying many chronic low-grade inflammatory diseases such as obesity, diabetes, inflammatory bowel disease, depression and Alzheimer’s disease [7,8,9,11,12]. These findings suggest that the gut microbiota and their metabolites, SCFAs (short-chain fatty acids), might be the missing link between “heating” foods and “shanghuo”. Modification of the gut microbiota by “heating” foods could be the mechanism underlying the ability of these foods to cause systemic low-grade inflammatory symptoms. In this study, we tested this hypothesis by investigating the effect of litchi, a typical “heating” food, on human gut microflora and low-grade inflammation indicators in a human flora-associated (HFA) mouse model. Litchi is a delicious fruit widely grown and consumed in many parts of America, Africa and Asia; it is known as a typical “heating” food in Chinese medicine, and thus, is a good representative of “heating” foods. The overall aim of this study was to understand the relationship between “heating” foods such as litchi and the gut microbiota by elucidating the mechanism by which such foods can cause low-grade inflammation symptoms or “shanghuo”. The findings of this study may provide a theoretical basis for the prevention and control of such disorders.

## 2. Materials and Methods

### 2.1. Material

#### 2.1.1. Experimental Animals

Specific pathogen-free (SPF), 6-week-old female C57BL/6J mice were purchased from Beijing Huafukang Biotechnology Co., LTD. (Beijing, China); the license batch number of the animals was SCXK (Beijing) 2016-0002. The animal experiments were conducted strictly in accordance with Guangdong Ocean University’s regulations on animal experimentation and the experimental procedures were approved by the University’s Ethics Committee on Experimentation with Animals (GDOU-20190724). The experimental animals were raised in Guangdong Ocean University’s Laboratory Animal Centre (License No. SYXK 2014-0053). The mice were fed an adequate supply of sterile water and Co60-irradiation sterilised feed with a nutrient composition consistent with the Chinese standard GB 14924.3-2001. The mice were housed under a controlled environment: temperature 20–25 °C, relative humidity 40–70%, pressure gradient 20–50 Pa, one-way flow of fresh air, and 12 h light–dark cycle. The water bottles, cage and pad materials used were sterilised by high-pressure steam. The water bottles and pad materials were changed three times per week.

#### 2.1.2. Preparation of Crude Litchi Extracts

Fresh litchi (*Litchi chinensis* Sonn.) was bought in Liufu Litchi Ecological Farm Co., LTD. (Zhanjiang, China); 5 kg, stored at 4 °C.

Fresh litchi pulp was cut into small pieces and soaked in distilled water (1:20, g/mL, pH 8.0) at 85 °C for 4 h. The water extracts were filtered and concentrated to one-fifth of the initial volume in a vacuum evaporator at 55 °C. Then, the precipitates were spray-dried to obtain the litchi extracts. The chemical composition of the litchi extracts was 68.78% water-soluble total sugar, 12.32% uronic acid and 3.56% protein [13].

#### 2.1.3. Materials, Chemicals and Reagents

Neomycin sulphate was obtained from Beijing Jintai Hongda Biotechnology Co., LTD. (Beijing, China); vancomycin (purity ≥ 98%) from Hefei Bomei Biotechnology Co., LTD. (Hefei, China); microplate quantitative chromogenic matrix limulus kit from Xiamen Limulus Reagent Biotechnology Co., LTD. (Xiamen, China); analytical grade acetic acid (AA), propionic acid (PPA), N-butyric acid (NBA), isobutyric acid (IBA), isovaleric acid (IVA), N-valeric acid (NVA) and N-caproic acid (NHV) from Shanghai Macklin Reagent Biotechnology Co., LTD.; and mouse TNF-α, IFN-γ, IL-1β and IL-6 ELISA kits from Neobioscience Technology Co., LTD. (Shenzhen, China).

### 2.2. Treatment of Experimental Animals

#### 2.2.1. Raising and Grouping of Human Faecal-Associated Mice

The raising of human faecal-associated (HFA) mice was performed according to the procedure of Wang et al. (2011) with minor modifications [14]. Briefly, the experimental mice were firstly acclimated for one week under the feeding conditions described above to ensure that they adapted to the feeding environment and reached the same baseline gut microbiota. After acclimation, the mice were gavaged with a mixture of antibiotics (vancomycin 400 mg/kg·d^−1^, neomycin 400 mg/kg·d^−1^ and metronidazole 400 mg/kg·d^−1^) for three days to obtain germ-free mice.

Fresh faeces were collected from a healthy volunteer (male, 19-years-old, without digestive tract or metabolic diseases and had not taken antibiotics in the previous three months); the obtained sample was the first bowel movement in the morning. Under the condition of anaerobic asepsis, the mass was measured, and 0.1 mol/L phosphate buffered saline (PBS) was added to dilute the content to a mass/water ratio of 1:9. The mixture was stirred to break up the faecal mass and then vortexed for 2 min to obtain a homogenous suspension. The suspension was stood for 10 min, and then the supernatant was collected as the human faecal microbial suspension. Twelve germ-free mice, obtained as described above, were gavaged with 0.3 mL of the faecal suspension, once every other day, for 3 weeks to allow the microbiota to colonize the intestinal tract of the mice. These mice were donated as HFA mice [14].

The 12 HFA mice were randomly divided into four groups with three mice in each group. The first three groups were gavaged daily with litchi powder solutions at a concentration of 400 mg/kg·d^−1^, 800 mg/kg·d^−1^ or 1600 mg/kg·d^−1^ alongside normal feeding for seven days; these mice were denoted as the low-, medium- and high-dose groups, respectively. The fourth group was gavaged with sterile water instead of litchi solution as a control.

#### 2.2.2. Collection of Mouse Blood and Faecal Samples

Mouse blood (about 1 mL) collected by eyeball extirpating was centrifuged at 1200× *g* and 4 °C for 5 min. The supernatant was removed, and the serum samples were stored at −80 °C until use. For the collection of faecal samples, mice were massaged on the abdomen, and fresh faecal particles were collected into sterile centrifuge tubes. The samples were immediately placed in an ice bath before being stored at −80 °C until use.

### 2.3. Measurement of Inflammatory Markers

The inflammatory markers TNF-α, IFN-γ, IL-1β and IL-6 in the mouse serum samples were measured by their respective ELISA kits, according to the manufacturer’s instructions. The concentration of lipopolysaccharides (LPS) was determined by a microplate quantitative chromogenic matrix limulus kit, according to the manufacturer’s instructions.

### 2.4. Gut Microbiota DNA Extraction

Total gut bacterial genomic DNA was extracted from the faecal samples using a PowerMax (stool/soil) DNA isolation kit (MoBio Laboratories, Carlsbad, CA, USA), following the manufacturer’s instructions, and was stored at −20 °C prior to further analysis. The quantity and quality of extracted DNA were measured using a NanoDrop ND-1000 spectrophotometer (Thermo Fisher Scientific, Waltham, MA, USA) and agarose gel electrophoresis, respectively.

### 2.5. 16S rDNA Amplicon Pyrosequencing

PCR amplification of the bacterial 16S rRNA gene V4 region was performed using the forward primer 515F (5′-GTGCCAGCMGCCGCGGTAA-3′) and the reverse primer 806R (5′-GGACTACHVGGGTWTCTAAT-3′). Sample-specific paired-end 6-bp barcodes were incorporated into the TrueSeq adaptors for multiplex sequencing. The PCR components included 25 μL of Phusion High-Fidelity PCR Master Mix, 3 μL (10 uM) of each forward and reverse primer, 10 μL of DNA template, 3 μL of DMSO, and 6 μL of ddH2O. Thermal cycling consisted of initial denaturation at 98 °C for 30 s, followed by 25 cycles consisting of denaturation at 98 °C for 15 s, annealing at 58 °C for 15 s, and extension at 72 °C for 15 s, with a final extension of 1 min at 72 °C. PCR amplicons were purified with Agencourt AMPure XP Beads (Beckman Coulter, Indianapolis, IN) and quantified using a PicoGreen dsDNA Assay kit (Invitrogen, Carlsbad, CA, USA). After the individual quantification step, amplicons were pooled in equal amounts, and pair-end 2 × 150 bp sequencing was performed using the Illlumina NovoSeq6000 platform at GUHE Info Technology Co., Ltd. (Hangzhou, China).

### 2.6. Sequence Analysis

The Quantitative Insights Into Microbial Ecology (QIIME, v1.9.0) pipeline was employed to process the sequencing data, as previously described [15]. Briefly, raw sequencing reads with exact matches to the barcodes were assigned to respective samples and identified as valid sequences. The low-quality sequences were filtered through the following criteria [16]: sequences that had a length of <150 bp, sequences that had an average Phred score of <20, sequences that contained ambiguous bases, and sequences that contained mononucleotide repeats of >8 bp. Paired-end reads were assembled using Vsearch V2.4.4 (–fastq_mergepairs--fastq_minovlen 5). Operational taxonomic units (OTUs) were selected using Vsearch V2.4.4 using Dereplication (–derep_full length), cluster (–cluster_fast, --id 0.97) and detection of chimeras (–uchime_ref) [17]. A representative sequence was selected from each OTU using the default parameters. OTU taxonomic classification was conducted by VSEARCH by searching the representative sequences against the Greengen database.

An OTU table was further generated to record the abundance of each OTU in each sample and the taxonomy of the OTUs. OTUs containing less than 0.001% of total sequences across all samples were discarded. To minimise the differences in sequencing depth across the samples, an averaged, rounded, rarefied OTU table was generated by averaging 100 evenly resampled OTU subsets under 90% of the minimum sequencing depth for further analysis.

### 2.7. Bioinformatics Analysis

Sequencing data analyses were mainly performed using the QIIME and R packages (v3.2.0). OTU-level alpha diversity indices, including the ACE metric (abundance-based coverage estimator), PD whole_tree, the Shannon diversity index and the Simpson index were calculated using the OTU table in QIIME.

OTU-level ranked abundance curves were generated to compare the richness and evenness of OTUs among samples. Beta diversity analysis was performed to investigate the structural variation in the microbial communities across the samples using UniFrac distance metrics [18,19], and was visualised via principal coordinate analysis (PCoA) and non-metric multidimensional scaling (NMDS) [20].

PCoA was conducted based on the genus-level compositional profiles [20]. A Venn diagram was generated to visualise the shared and unique OTUs among the samples or groups using the R package “VennDiagram”; this analysis was based on the occurrence of OTUs across samples/groups regardless of their relative abundance [21]. Taxa abundances at the phylum, class, order, family, genus and species levels were statistically compared among samples or groups by the Kruskal test using the R stats package. Microbial functions were predicted by PICRUSt (phylogenetic investigation of communities by reconstruction of unobserved states) based on high-quality sequences [22]. The output file was further analysed using the Statistical Analysis of Metagenomic Profiles (STAMP) software package v2.1.3 [23]. FAPROTAX is a database that maps prokaryotic clades (e.g., genera or species) to established metabolic or other ecologically relevant functions [24].

### 2.8. Faecal Short-Chain Fatty Acids (SCFA) Analysis by Gas Chromatography–Mass Spectrometry (GC-MS)

All sample processing procedures were performed at 4 °C to minimise the loss of volatile metabolites, unless stated otherwise. To extract polar metabolites for GC-MS analysis, faecal samples were thawed on ice. About 50 mg of each sample was obtained and added to 100 μL of 15% phosphoric acid. Then, 125 μg/mL of internal standard (isohexanoic acid) solution, 100 μL and diethyl ether 900 μL homogenate for 1 min. The sample was then centrifuged at 4 °C, 15,300× *g* for 10 min. The mixed solution was filtered with 0.22 μm organic microporous membrane, and the supernatant was obtained and tested on an Agilent 5975C-7890A machine.

The injection port temperature was 270 °C and the flow rate of nitrogen, which was supplied as the carrier gas, was 3 mL/min. The initial column temperature was 50 °C and was increased by 10 °C/min to 100 °C, then maintained for 1 min, increased by 5 °C/min to 150 °C and then maintained again for 5 min before finally increasing to 250 °C at 20 °C/min. The injected sample volume for the GC-MS analysis was 2 μL and the temperature of the FID was 280 °C. The calibration curve method was used for the quantitative determination of faecal SCFAs.

### 2.9. Hematoxylin and Eosin (H&E) Staining of Intestine Tissues

At the end of the experimental period, the mice were sacrificed and dissected. The last 5 cm of the intestine was cut off and then sliced longitudinally. The specimens were immediately immerged in 10% formalin and then stained with H&E. Sections of the specimens were examined under a light microscope and assessed for histological damage. At least three sections from each animal were examined. The prepared glass slides carrying samples were observed under a microscope (10 × 10). The numbers of goblet cells, villi length and crypt depth in a fixed area were counted. A total of five horizons were selected for each sample. Then the averages for each sample and for each group were calculated.

### 2.10. Statistical Analysis

The results are presented as the mean and standard error of the mean (SEM). Statistical analysis was performed using SPSS 21.0 (SPSS, Chicago, IL, USA). Comparisons between groups were made with one-way analysis of variance (ANOVA) followed by the LSD test, with *p* < 0.05 considered statistically significant.

## 3. Results

### 3.1. Analysis of the Similarity between the Intestinal Flora of Human Flora-Associated (HFA) Mice Model and Volunteers at the Phylum and Genus Levels

**Figure 1:**
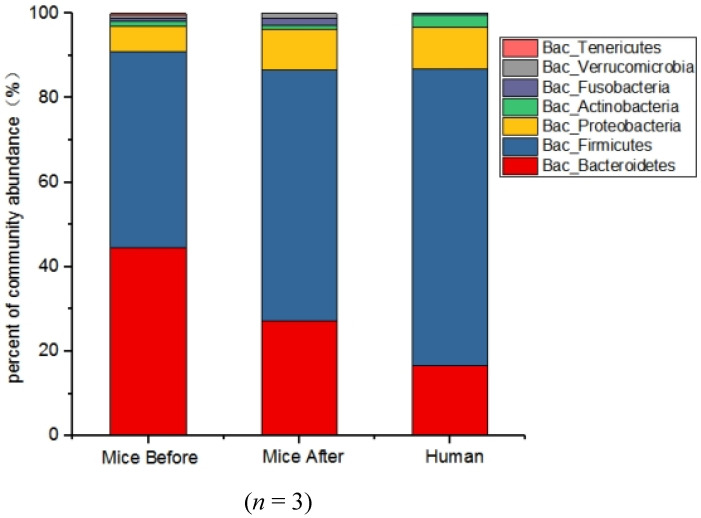
The comparison of gut flora composition at phylum level between human flora-associated (HFA) mice model and volunteer. (Mice Before: blank control mice, Mice After: HFA mice, and Human: gut microbiota of volunteer).

We performed 16SrDNA sequencing analysis on feces of normal mice, feces of humanized mice and feces samples of volunteers. As shown in Figure 1, the three groups of samples were mainly composed of *Firmicutes* and *Bacteroidetes*. Compared with the volunteers, the proportion of *Bacteroides* in the intestines of antibiotic-treated mice increased by 9.71% and *Firmicutes* decreased by 10.05% after being gavaging with the feces of the volunteers. *Bacteroides/Firmicutes* ratio can be used as an indicator to measure the composition of intestinal flora. The *Bacteroides/Firmicutes* ratio in blank control mice group was 0.968, and the *Bacteroides/Firmicutes* ratio was 0.482 at the phylum level in the HFA mice group. The ratio of *Bacteroides* to *Firmicutes* in the feces of the volunteers was 0.267, indicating that the intestinal microflora of the transplanted mice was close to the composition of the feces of the volunteers.

According to Table 1, the relative abundance of *Bacteroides* in the gut microbiota of mice after recombination is still the most dominant. Compared with the abundance of *Bacteroides* in mice before recombination, the relative abundance of *Bacteroides* after recombination is nearly twice as high as 16.2%. The proportion is very close to the proportion of bacteroidetes in humans. The number of *Bifidobacterium*, *Parabacteroides*, *Clostridium* and *Akkermansia*, which are important beneficial bacteria to human body, increased 2–3 times compared with that before recombination. The relative abundance of *Eggerthella* increased by nearly 0.3% from 0. The percent of community abundance of *Prevotella*, *Desulfovibrio*, *Ruminococcus* has reduced compared with the control group, and the percentage of genera of HFA mice group is close to that in volunteer feces.

### 3.2. Effect of Litchi on Inflammatory Factors in HFA Mice

**Figure 2:**
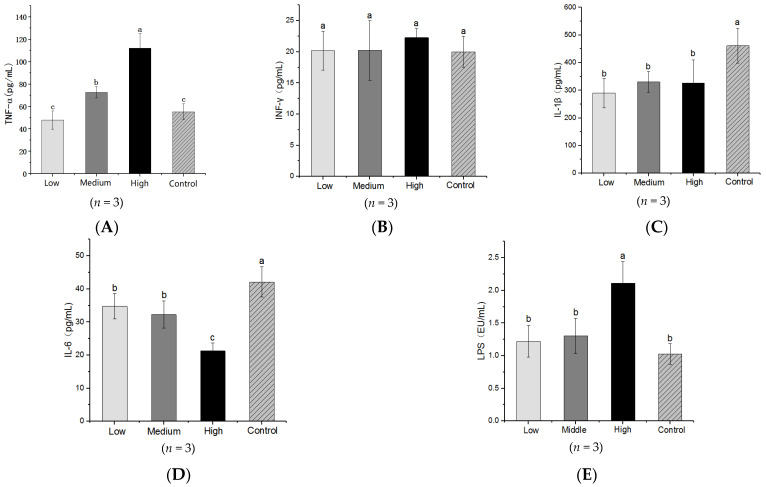
Effects of litchi doses on the four inflammatory markers and lipopolysaccharides (LPS) levels in the plasma of mice submitted to different treatments: (**A**) TNF-α, (**B**) INF-γ, (**C**) IL-1β, (**D**) IL-6 and (**E**) LPS. Low, middle and high denote mice that received low, middle and high doses of litchi gavage; control mice did not receive litchi gavage. Data are means ± SE. Data with different letters (a,b,c) are significantly different in different group (*p* < 0.05) according to the ANOVA statistical analysis.

Gavaging HFA mice with litchi caused significant changes to the four inflammatory markers, TNF-α, IFN-γ, IL-1β and IL-6; however, the effects on the markers were quite different. Litchi exhibited a dose-dependent effect on TNF-α. The level of TNF-α in the low litchi dose group was not significantly different from that of the control, but the levels in the medium- and high-dose mice was significantly higher than the control (*p* < 0.05) (Figure 2A). The level of TNF-α in the high litchi dose group was more than two times higher than that of the control mice group. Litchi gavage of HFA mice caused significant (*p* < 0.05) decreases in the IL-1β concentration compared with the control, for all three dose groups; however, the three dose groups did significantly differ from one another (*p* > 0.05) (Figure 2C). The IL-6 level in the high litchi dose group was significantly lower than that in the control group, and lower than the low- and medium-dose groups (*p* < 0.05); however, the latter two groups did not significantly differ from one another (*p* < 0.05) (Figure 2D). No significant difference (*p* > 0.05) in the IFN-γ level was observed between the litchi-gavaged HFA mice and the control mice (Figure 2B).

The plasma LPS levels in the different groups of mice are shown in Figure 2E. The LPS levels in the low- and medium-dose litchi groups were higher than the control, but the differences were not statistically significant. However, the plasma LPS concentration of the litchi high-dose group was 106.12% higher than the control group, and this was statistically significant (*p* < 0.05).

### 3.3. Effect of Litchi Intervention on the Diversity of the Gut Microbiota in Mice

#### 3.3.1. Effect of Litchi Intervention on Alpha Diversity

Table 2 shows goods coverage values in all the experimental groups, with coverage above 99.00%. The value in the control group was above 99.88%. This demonstrates that the data volume for sequencing was sufficient, and the sequencing results are an appropriate representation of the sample. As shown in Table 2, both the Shannon and Simpson indices in the low-, medium- and high-dose litchi groups were significantly (*p* < 0.05) higher than that in the control group (*p* < 0.05). Furthermore, the Chao1 and Ace indices for the three litchi groups were about 39–80% and 32–82% higher than the control, respectively. These results demonstrate that both the abundance and diversity of the gut flora in the litchi groups were significantly higher than the control mice.

#### 3.3.2. Effect of Litchi Intervention on Operational Taxonomic Unit (OTU) of Mouse Gut Microbiota

**Figure 3:**
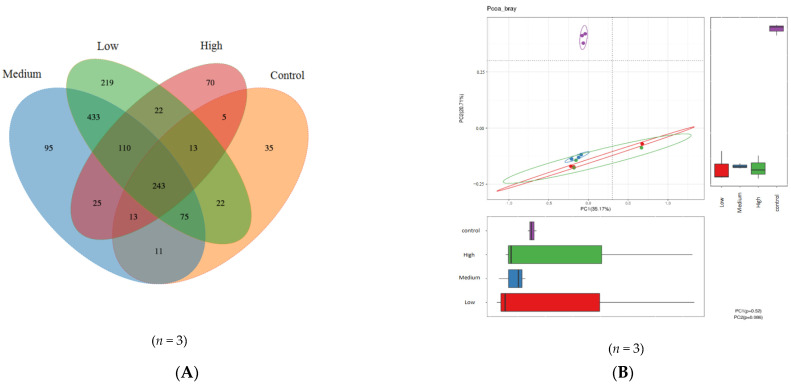
Effect of litchi intervention on the diversity of mouse gut microbiota. (**A**) Comparison of OTUs of gut flora between litchi-gavaged HFA mice and control mice. The Venn diagram shows the common and unique OTUs in the different groups. The number in the core represents the OTUs common to all groups and the numbers on the non-overlapping areas represent the total OTUs of each group minus the number of shared OTUs. (**B**) Principal coordinate analysis (PCoA) of the gut microbiota of mice in the litchi experimental groups and control group.

A Venn diagram was plotted (Figure 3A) to show the common and unique OTU among the four groups of mice (low, medium and high dose of litchi, and control). Of the >1300 observed OTUs, 243 were common to all groups. The common number of OTUs between the control group and the low-, medium- and high-dose group was 353, 342 and 274, respectively. In total, 784 specific OTUs were found for the low-dose group while 64 were found for the control group; 663 specific OTUs were found for the medium-dose group while 75 were found for the control group; 227 specific OTUs were found for the high-dose group while 143 were found for the control group. These results indicate that litchi intervention caused significant increases in the species richness (*p* < 0.05) and greater increases occurred with low and medium doses of litchi intervention compared to the high dose.

#### 3.3.3. Effect of Litchi Intervention on the β-Diversity of the Gut Microbiota in Mice

β-diversity estimates of the gut microflora of the different mouse groups were calculated by computing unweighted UniFrac distances and were visualised by PCoA (Figure 3B). The distances between the control and all three litchi dose groups were very large, while the distances between the three litchi dose groups were quite small. The results indicate that litchi intervention at all three doses caused significant changes in the β-diversity of the gut flora of mice compared with the control mice (*p* < 0.05).

### 3.4. Effect of Litchi Intervention on the Gut Flora of Mice at the Phylum and Genus Levels

**Figure 4:**
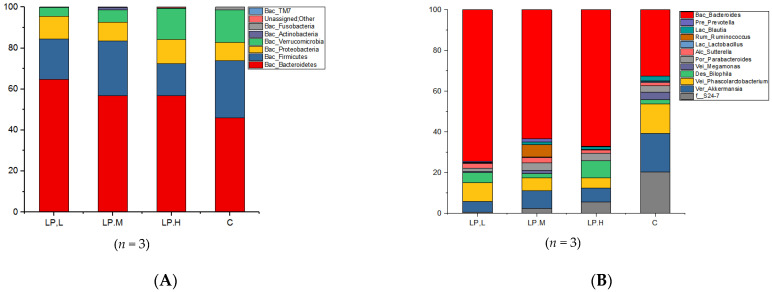
The composition of the gut microbiota of mice in the litchi-gavaged and control groups at the (**A**) phylum level and (**B**) genus level.

The gut microbiota of the mice was analysed at the phylum level (Figure 4A). The gut microbiota of all the mouse groups was dominated by four phyla, Bacteroidetes, Firmicutes, Proteobacteria and Verrucomicrobia, which together accounted for more than 98% of the gut microflora. Litchi intervention significantly altered the composition of the gut microbiota. The abundance of Bacteroidetes and Proteobacteria was increased by 27–40% and 1–38%, respectively, in the litchi intervention groups compared with the control group, with greater increases in the low litchi dose group. On the other hand, litchi intervention caused a decrease in the abundance of Firmicutes and Verrucomicrobia by 11–47% and 11–71%, respectively, compared with the control group. The abundance of two minor phyla, Actinomycete and Fusobacteria, also changed significantly as a result of litchi intervention, with the former increasing by 4.4–36.4 times while the latter decreased by 8.7–297.9 times, compared with the control group.

Figure 4B compares the genus-level gut microbiota of mice in the different treatment groups (Figure 4B). *Bacteroides* was the most dominant genus in all mouse groups, but several other genera, including *Akkermansia, Bilophila* and *Phascolarctobacterium,* were also present in significant proportions. Gavaging of mice with litchi had a significant impact on the composition of the gut microflora at the genus level. For some genera, litchi intervention resulted in significant increases in abundance. This included *Bacteroides*, whose proportion in the gut microflora was 61.39%, 42.92% and 49.39% in the low-, medium- and high-dose litchi groups, respectively, all of which were significantly higher than the proportion in the control group (26.55%). Moreover, the proportion of *Bilophila* was 3.4 times higher in the high-dose litchi group than in the control group. Other genera, such as *Prevotell,* also exhibited significant increases in abundance as a result of litchi intervention. *Prevotell* was undetected in the control mice, but accounted for 0.56%, 1.07% and 0.24% of the gut microflora, respectively, in the low-, medium- and high-dose litchi groups. On the other hand, the abundance of some genera, including *Akkermansia Phascolarctobacterium, Megamona* and *Lactobacillus,* generally decreased as a result of litchi intervention. The abundance of *Akkermansia* was 3.4, 2.9 and 3.3 times lower and the abundance of *Phascolarctobacterium* was 1.5, 2.8 and 3.1 times lower in the low-, medium- and high-dose groups, respectively, compared to the control group (*p* < 0.001). The abundance of *Megamonas* decreased by 99.3 and 7.1 in the low- and high-dose groups (but increased by 72.9% in the medium-dose group), while the abundance of *Lactobacillus* decreased by 4.7 and 2.1 in the low- and high-dose groups (but increased by 74.0% in the medium-dose group), respectively, compared with the control group (*p* < 0.001).

### 3.5. Functional and Metabolic Phylogenetic Investigation of Communities by Reconstruction of Unobserved States (PICRUSt) Analysis and Faportax Analysis of the Gut Microbiota

**Figure 5:**
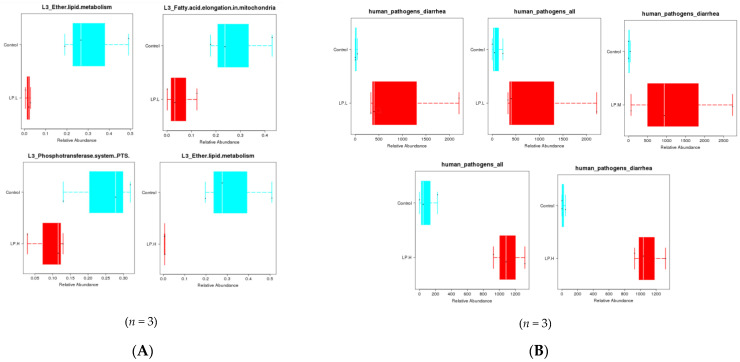
(**A**) Phylogenetic investigation of communities by reconstruction of unobserved states (PICRUSt) analysis and (**B**) Farportax function analysis of the differences between the metabolic pathways of the litchi intervention and control groups.

PICRUSt analysis of the metabolic pathways showed that with low-dose litchi intervention, ether lipid metabolism and fatty acid elongation in mitochondria were down-regulated (Figure 5A). With the medium-dose litchi intervention, the synthesis of restriction enzymes for the synthesis of secondary metabolic functional proteins as well as lipid metabolism were up-regulated, but ether lipid metabolism was down-regulated. With the high-dose litchi intervention, the phosphate transferase system and ether lipid metabolism in lipid metabolism were down-regulated (*p* < 0.05).

Figure 5B shows the results of the Farportax functional analysis. The low-, medium- and high-dose litchi intervention led to significant up-regulation of the pathogenic bacteria related to human diarrhoea in the gut microflora of mice, while medium and high doses of litchi intervention also resulted in significant increases in the total number of human pathogenic bacteria.

**Figure 6:**
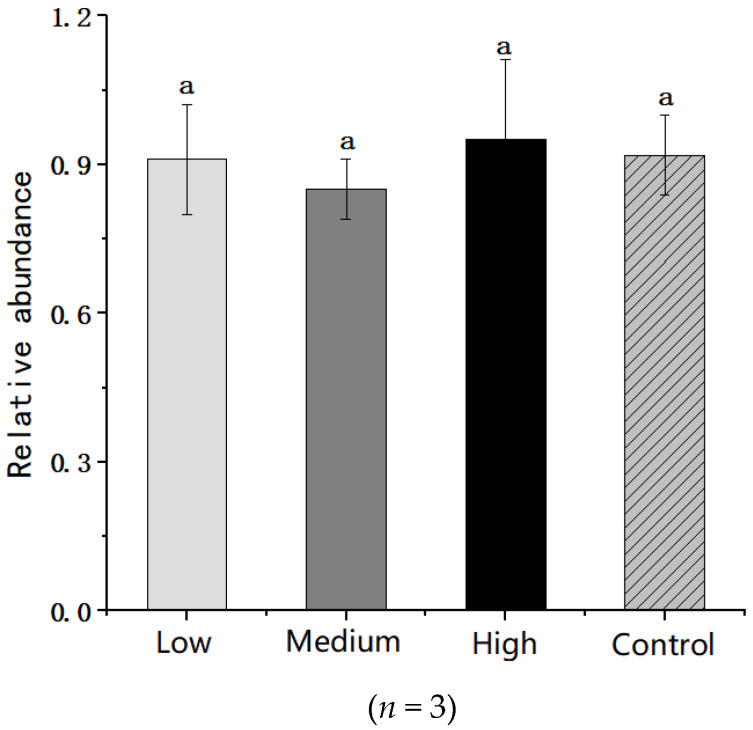
Comparison of the abundance of gram-negative bacteria in the gut microbiota between the litchi invention and control groups. Data with same letters (a) are no significantly difference in different groups (*p* > 0.05) according to the ANOVA statistical analysis.

The abundance of Gram-negative bacteria in the gut microflora was also analysed but no significant differences were observed between the control and the three litchi invention groups (*p* > 0.05) (Figure 6).

### 3.6. Effect and Correlation between Litchi Intervention and SCFAs in the Intestines of Mice

Table 3 shows the contents of SCFAs in the faecal samples of mice in the three different dose groups and the control group. The contents of AA, PPA, NBA and IBA in the high-dose litchi group were significantly (*p* < 0.05) lower than those in the control group. The contents of PPA and IBA in the mid-dose litchi group were significantly (*p* < 0.05) lower than those in the control group. These results demonstrate that high-dose litchi gavage can significantly decrease the contents of several SCFAs in the intestine.

**Figure 7:**
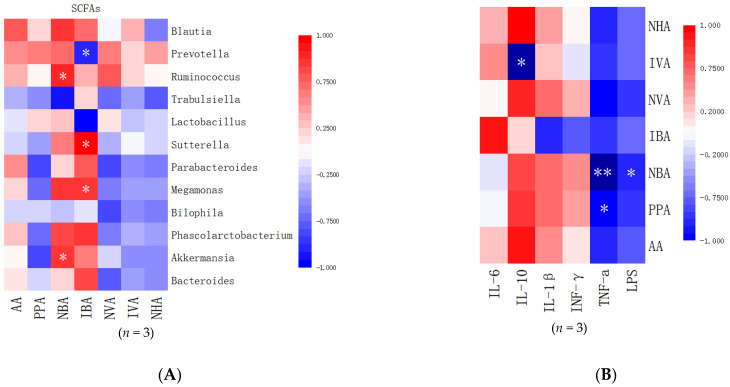
Correlation analysis between short-chain fatty acids, gut microbiota and inflammatory factors. (**A**) Correlation between the gut microbiota and SCFAs. (**B**) Correlation between inflammatory factors and SCFAs. Pearson correlation analysis: * correlation is significant at the 0.05 level (two-tailed), ** correlation is significant at the 0.01 level (two-tailed). AA: acetic acid, PPA: propionic acid, NBA: N-butyric acid, IBA: isobutyric acid, IVA: isovaleric acid, NVA: N-valeric acid, NHV: N-caproic acid.

Figure 7A shows the correlations between the gut microbiota and SCFAs. *Akkermansia* and *Ruminococcus* were positively correlated with NBA (r = 0.878, *p* = 0.012; r = 0.912, *p* = 0.044, respectively). In addition, *Megamonas* and *Sutterella* were positively correlated with IBA (r = 0.893, *p* = 0.039; r = 0.962, *p* = 0.038, respectively) while *Prevotella* was negatively correlated with IBA (r = −0.932, *p* = 0.029).

Figure 7B shows the correlations between the inflammatory factors and SCFAs. NBA and PPA were negatively correlated with TNF-α (r = 0999, *p* = 0.003; r = 0.985, *p* = 0.020, respectively). NBA was negatively correlated with LPS (r = 0.976, *p* = 0.018) while IVA was negatively correlated with IL-10.

### 3.7. Effect of Litchi Intervention on Intestinal Mucosal Structure

**Figure 8:**
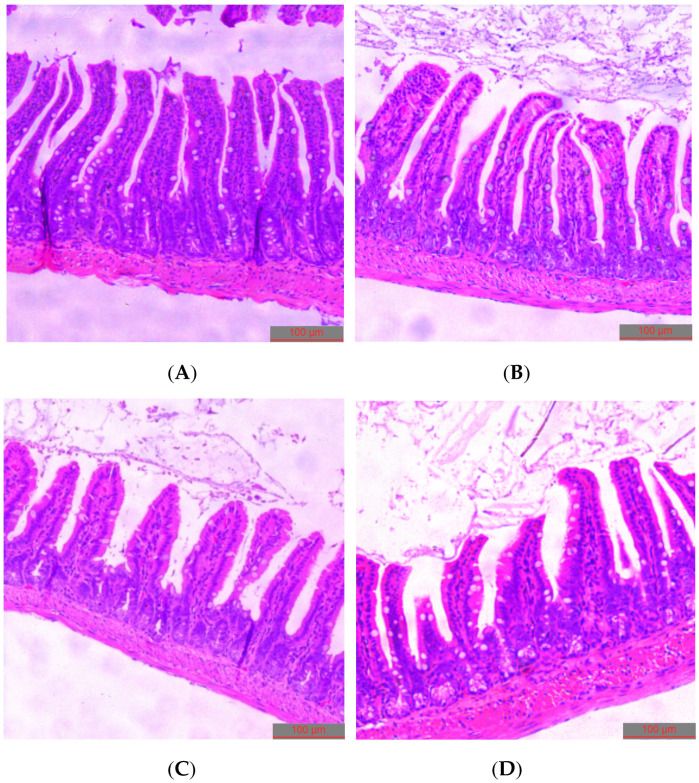
Histological appearance of intestinal tissue cross-sections of mice submitted to different treatments. (**A**) Control, (**B**) low-dose litchi intervention, (**C**) mid-dose litchi intervention, (**D**) high-dose litchi intervention.

**Figure 9:**
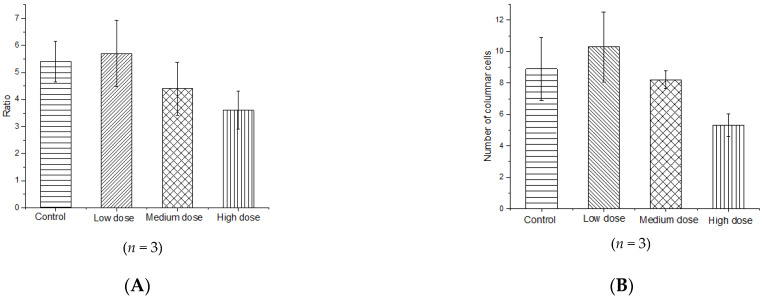
(**A**) Ratio of intestinal villi length to crypt depth and (**B**) mean number of goblet cells in visual field.

Histological analysis of the intestinal tissue cross-sections of mice (Figure 8) revealed that litchi intervention had a notable impact on the colon tissue structure. In the control and low-dose litchi groups, the epithelial cells were whole and tightly packed, and the structural gap in the connective complex between the epithelial cells was normal. However, in the mid- and high-dose litchi groups, the colonic mucosal epithelial cells were loose and not tightly packed, the gap between the epithelial connective complex was widened, and the epithelial cells had sparse microvilli. The mid- and high-dose litchi groups also showed some degree of pathological lesions, including superficial epithelial damage and thickening of the muscularis propria. Both the ratio of intestinal villi length to crypt depth and mean number of goblet cells in high dose group show significant decreases compared to the control group (Figure 9).

## 4. Discussion

This study investigated the interrelations between foods, systematic low-degree inflammation and the gut microbiome with the aim of elucidating the mechanism by which foods such as litchi, which is a typical “heating” food in Chinese medicine, affect human health. To achieve this goal, a mouse model was constructed. In the model, the gut was first sterilised by the administration of antibiotics and then transplanted with a faecal microbial suspension from a healthy human adult. When the mice were given high-dose litchi extract, the serum levels of TNF-α, a typical inflammatory marker, and LPS were more than two times higher than those of the control mice. It is generally believed that when the serum level of TNF-α increases by 2–4 times the normal level, systemic low-grade inflammation occurs [25]. Thus, a clear link between excessive consumption of litchi and systematic low-degree inflammation was found. Litchi intervention also caused a significant decrease in serum IL-1β and IL-6 levels. Increased production of IL-1β has been reported to be associated with several autoinflammatory disorders [26] while IL-6 is linked with the pathogenesis of a number of diseases such as rheumatoid arthritis, cancer, multiple sclerosis, anaemia, inflammatory bowel disease, Crohn’s disease and Alzheimer’s disease [27]. In this regard, the consumption of litchi may also confer a number of health benefits through the lowering of IL-1β and IL-6 levels in the body. Thus, the relationship between litchi invention and health appears to be complex and may be related to the intervention dosage.

Inflammation is a double-edged sword for the health of the body. Moderate inflammation is important for the body’s own defence, but excessive or persistent systemic inflammation can have adverse effects, leading to a variety of chronic diseases [9,10]. There is growing evidence that disorder of the gut microbiota (dysbacteriosis) may play a key role in the development of chronic inflammatory diseases [7,9,12,28,29]. For example, Ridaura et al. (2013) and Dao et al. (2016) found that the proportion of Firmicutes to Bacteroides and the abundance of Oscillibacter, Clostridium and Akkermansia muciniphila in the gut microflora were associated with systemic inflammation induced by obesity [28,29]. Rosen et al. (2017) reported that inflammatory bowel disease was related to decreased abundance of microbes with anti-inflammatory potential (such as Bifidobacterium and Lactobacillus) and increased abundance of pathogenic bacteria (such as Staphylococcus aureus and Clostridium difficile) [12]. However, there have been relatively few studies of the role of food in disorder of the gut microbiota and its association with systemic low-grade inflammation. Most studies have focused on well-known unhealthy foods or food components such as a high-fat diet, white bread, saturated fat, emulsifiers and other ingredients [10,29,30,31,32]. This study is among the first to examine the possible effects of seemingly healthy foods such as litchi, a delicious fruit, on dysbacteriosis and low-grade systematic inflammation. The results demonstrated that litchi intervention significantly increased the diversity and species richness of the gut microbiota of mice, but the effect was much greater with low-dose litchi than high-dose litchi. Litchi intervention also significantly altered the composition of the gut microflora both at the phylum and genus levels. With litchi intervention, the abundance of Phascolarctobacterium, Akkermansia, Megasomonas and Lactobacillus generally decreased, while the abundance of Prevotella and Bacteroides increased. These effects were most obvious in the high-dose group. Phascolarctobacterium, Akkermansia, Megasomonas and Lactobacillus have all been reported to reduce inflammation and play a beneficial role in the control of inflammation [33]. A significant reduction in the abundance of all four species is likely to an increase the risk of inflammation. Furthermore, it has been suggested that an increase in the abundance of Bilophila and Prevotella may also lead to increased risk of intestinal inflammation [34,35]. Thus, the decreases in the abundance of Megasomonas and the increase in Bacteroides and Bilophilia, especially in the high-dose group, might be linked with the systemic low-grade inflammation caused by overconsumption of litchi.

There has been considerable research into the mechanisms by which dysbacteriosis of the gut microbiota induces systemic low-grade inflammation, and the structural integrity of the intestinal barrier is generally believed to play an important role in this process [36]. As metabolites of intestinal flora, SCFAs have been shown to improve the systemic inflammatory response and maintain intestinal homeostasis [37]. It has been reported that SCFAs can induce neutrophil and regulatory T cell responses to inhibit inflammation through G protein-coupled receptor activation and histone deacetylase inhibition pathways [38]. In this study, high-dose litchi gavage was found to significantly reduce the contents of multiple SCFAs such as AA, PPA and NBA, indicating that the ability to inhibit inflammation was reduced.

There are also reports that SCFAs, as important metabolites of intestinal flora, maintain the integrity of the intestinal barrier by providing energy to intestinal epithelial cells and regulating the tight connection between intestinal mucosal cells [37]. In this study, high-dose litchi gavage significantly reduced the proportion of microbiota-produced SCFAs, such as Megamonas and Lactobacillus. Lactobacillus can promote the production of PPA and NBA in the intestine and enhance the uptake of SCFAs in intestinal epithelial cells by increasing adhesion to the intestine [39]. Lactobacillus can also provide energy for cell proliferation, promote the proliferation and differentiation of intestinal epithelial cells, and maintain the integrity of the intestinal barrier [40]. Megamonas was positively correlated with IBA in this study. IBA can reportedly enhance the expression of tight junction proteins and stabilise the intestinal mucosal barrier to limit the transfer of bacteria and other microorganisms from the gut to the bloodstream, thereby reducing the body’s inflammatory response [38]. An intestinal biopsy indicated that litchi intervention, especially with the medium and high doses, caused visible increases in the gap of the intestinal epithelial junction, in addition to a number of other pathological changes to epithelial tissues, which may be related to the decrease in SCFAs in the intestine. This may allow entry of intestinal bacteria and their products, further inducing an inflammatory response. Furthermore, with the high litchi dose, diarrhoea-related pathogenic bacteria, as well as the total number of pathogenic bacteria in the gut microbiota of mice were significantly up-regulated, which could increase the risk of intestinal mucosal inflammation, thus affecting the integrity of the intestinal mucosal barrier. These results are consistent with the increased concentration of inflammatory factors such as TNF-α in the serum of the high-dose litchi group. In addition, litchi intervention was found to significantly down-regulate the phosphatase transferase system and lipid metabolism capacity of the gut microbiota, indicating that the litchi intervention reduced the energy conversion efficiency of the gut microbiota. The litchi extracts were primarily composed of water-soluble total sugar, uronic acid and protein; the reduced energy conversion efficiency of the intestinal flora would cause an increase in the intestinal absorption of sugar, which may have health implications. However, the current results cannot determine whether this is detrimental or beneficial to health. Previous studies have shown that the production of SCFAs is related to the structural characterization of polysaccharides, such as monosaccharide compositions, glycosidic linkage, molecular weight, branch chain, particle size, solubility and viscosity [41]. How litchi extract affects the formation of SCFAs in the intestine and what exact component makes it work requires further study.

## 5. Conclusions

This preliminary study demonstrated that litchi intervention in HFA mice can significantly alter the gut microflora, induce inflammation and cause damage to the intestinal mucosal tissues of mice. Litchi intervention, especially at a high dose, caused a more than two-fold increase in the serum TNF-α and LPS levels. The intervention led to significant decreases in the abundance of microorganisms that are associated with inflammation reduction and increases in those linked to elevated inflammation. The litchi intervention also produced significant increases in the total number of pathogenic bacteria related to diarrhoea and other diseases. Furthermore, the medium- and high-dose litchi intervention reduced the concentrations of a variety of SCFAs in the intestinal tract, leading to a decline in the body’s inflammatory suppression function. Reduced SCFA concentration also resulted in weakening the intestinal epithelial cell junction and the intestinal mucosal barrier function. Thus, this study provides preliminary evidence that the Chinese medicine notion that litchi, as a “heating” food, when consumed excessively can lead to low-degree systematic inflammation or “heating up”. This study also demonstrates that litchi-induced inflammation is linked to its ability to cause dysbacteriosis of the gut microbiota and weakening of the intestinal mucosal tissues. However, this study did not examine the components of litchi that are responsible for these changes in mice, which is a topic worthy of study in the future.

## Figures and Tables

**Table 1:** Comparison of the composition of the representative genus in the gut microbiota of the HFA mice and volunteers.

Genus	Mice Before	Mice After	Human
Bacteroides	8.92% ± 1.34% ^b^	16.2% ± 2.38% ^a^	18.6% ± 3.05% ^a^
Bifidobacterium	0.32% ± 0.05% ^c^	0.83% ± 0.16% ^b^	1.41% ± 0.16% ^a^
Parabacteroides	0.25% ± 0.02% ^c^	0.62% ± 0.09% ^b^	2.29% ± 0.29% ^a^
Clostridium	0.67% ± 0.13% ^c^	1.44% ± 0.31% ^b^	2.16% ± 0.33% ^a^
Akkermansia	0.91% ± 0.10% ^b^	1.90% ± 0.40% ^a^	1.78% ± 0.21% ^a^
Eggerthella	0% ± 0% ^c^	0.38% ± 0.05% ^b^	0.96% ± 0.10% ^a^
Prevotella	0.84% ± 0.21% ^a^	0.52% ± 0.07% ^b^	0.19% ± 0.04% ^c^
Desulfovibrio	1.41% ± 0.19% ^a^	0.93% ± 0.10% ^b^	0.26% ± 0.04% ^c^
Ruminococcus	0.59% ± 0.08% ^a^	0.12% ± 0.04% ^c^	0.25% ± 0.07% ^b^

Data are means ± SE. Data with different letters (a,b,c) are significantly different in different group (*p* < 0.05) according to the analysis of variance (ANOVA) statistical analysis.

**Table 2:** Effect of litchi on alpha diversity of the gut microbiota in mice.

Litchi Dose	Shannon	Simpson	Chao1
Low	5.475187 ± 0.16 ^a^	0.936068 ± 0.03 ^a^	959.0170 ± 36.40 ^b^
Medium	5.639477 ± 0.18 ^a^	0.954772 ± 0.03 ^a^	860.4412 ± 27.07 ^b^
High	5.703059 ± 0.21 ^a^	0.932552 ± 0.04 ^a^	1115.216 ± 39.15 ^a^
Control	4.181543 ± 0.13 ^b^	0.759437 ± 0.02 ^b^	616.6304 ± 16.23 ^c^

Data are means ± SE. Data with different letters (a,b,c) are significantly different in different group (*p* < 0.05) according to the ANOVA statistical analysis.

**Table 3:** The contents of SCFAs in the faecal samples of mice in the four groups.

Group	AA (μg/g)	PPA (μg/g)	NBA (μg/g)	IBA (μg/g)	NVA (μg/g)	IVA (μg/g)	NHV (μg/g)
Low-dose litchi	3082.82 ± 319.62 ^ab^	527.66 ± 58.01 ^ab^	24.17 ± 1.89 ^a^	81.19 ± 10.07 ^a^	12.18 ± 1.02 ^a^	9.15 ± 1.82 ^a^	2.51 ± 0.33 ^a^
Mid-dose litchi	2892.29 ± 266.09 ^ab^	485.75 ± 39.21 ^b^	23.71 ± 1.52 ^a^	58.40 ± 7.67 ^b^	9.74 ± 1.36 ^a^	9.84 ± 0.31 ^a^	2.10 ± 0.19 ^a^
High-dose litchi	2781.11 ± 78.12 ^b^	421.87 ± 28.98 ^b^	20.65 ± 1.20 ^b^	50.35 ± 6.34 ^b^	9.50 ± 1.89 ^a^	9.30 ± 0.63 ^a^	2.32 ± 0.26 ^a^
Control	3316.43 ± 212.39 ^a^	610.93 ± 52.81 ^a^	25.58 ± 1.92 ^a^	87.58 ± 10.29 ^a^	12.07 ± 3.00 ^a^	7.12 ± 2.15 ^a^	2.38 ± 0.31 ^a^

AA: acetic acid, PPA: propionic acid, NBA: N-butyric acid, IBA: isobutyric acid, IVA: isovaleric acid, NVA: N-valeric acid, NHV: N-caproic acid. Data are shown as the mean ± SD. Data with different letters (a,b,c) are significantly different in different group (*p* < 0.05) according to the ANOVA statistical analysis.

## Data Availability

Sequencing reads were deposited in the NCBI’s sequence read archive under accession number PRJNA648688. Further data are available from the corresponding author on reasonable request.

## Abbreviations

qPCR	Quantitative real-time polymerase chain reaction
ELISA	Enzyme-linked immunosorbent assay
LPS	Lipopolysaccharide
TNF	Tumor necrosis factor
IFN	Interferon
IL	Interleukin
